# Diagnostic Accuracy of Droplet Digital PCR and Amplification Refractory Mutation System PCR for Detecting EGFR Mutation in Cell-Free DNA of Lung Cancer: A Meta-Analysis

**DOI:** 10.3389/fonc.2020.00290

**Published:** 2020-03-03

**Authors:** Caichen Li, Qihua He, Hengrui Liang, Bo Cheng, Jianfu Li, Shan Xiong, Yi Zhao, Minzhang Guo, Zhichao Liu, Jianxing He, Wenhua Liang

**Affiliations:** China State Key Laboratory of Respiratory Disease and National Clinical Research Center for Respiratory Disease, The First Affiliated Hospital of Guangzhou Medical University, Guangzhou, China

**Keywords:** lung cancer, droplet digital PCR (ddPCR), amplification refractory mutation system PCR (ARMS-PCR), cell free DNA (cfDNA), epidermal growth factor receptor (EGFR)

## Abstract

**Background:** Epidermal growth factor receptor (EGFR) mutation testing in plasma cell-free DNA (cfDNA) from advanced lung cancer patients is an emerging clinical tool. This meta-analysis was designed to determine the diagnostic accuracy of two common PCR systems, droplet digital PCR (ddPCR) and amplification refractory mutation system PCR (ARMS-PCR), for detecting EGFR mutation in cfDNA.

**Materials and methods:** A systematic search was carried out based on PubMed, Web of science, Embase and the Cochrane library. Data from eligible studies were extracted and pooled to calculate the sensitivity, specificity, diagnostic odds ratio (DOR), area under the summary receiver-operating characteristic curve (AUROC), using tissue biopsy results as the standard method. Subgroup analyses were performed regarding EGFR mutation type, tumor stage, and EGFR-TKI treatment.

**Results:** Twenty-five studies involving 4,881 cases were included. The plasma testing sensitivity, specificity, DOR, and AUROC, compared with the matched tumor tissues, were 72.1%, 95.6%, 38.5, 0.89 for ddPCR, and 65.3%, 98.2%, 52.8, 0.71 for ARMS-PCR, respectively, through indirect comparison, significant differences were found in sensitivity (*P* = 0.003) and specificity (*P* = 0.007). Furthermore, significant difference was found in sensitivity between tumor stage subgroups (IIIB–IV subgroup vs. IA–IV subgroup) in ARMS-PCR (73.7 vs. 64.2%, *P* = 0.008), but not in ddPCR (72.5 vs. 71.2%, *P* = 0.756).

**Conclusions:** This study demonstrates that ddPCR and ARMS-PCR have a high specificity with a practical sensitivity for detecting EGFR mutation in cfDNA, which supports their application as a supplement or a conditional-alternative to tissue biopsy in clinical practice for genotyping. It seems that ddPCR has a higher sensitivity than ARMS-PCR, especially in early stages.

## Introduction

Lung cancer remains the most frequently diagnosed cancer and the leading cause of cancer-related mortality worldwide, with 85% of patients having non-small-cell lung cancer (NSCLC) (1–3). Fortunately, accurate gene analysis of epidermal growth factor receptor (EGFR) mutation in advanced NSCLC patients has provided them great opportunities to receive optimal treatments. Successful analysis of genotyping plays an important role in this process (4, 5). Conventionally, detection of EGFR mutation status in tumor tissue is the standard approach, which can be obtained by tissue biopsy or surgery (6). However, tissue samples are not always available or sufficient in quantity for genotyping. Furthermore, tissue biopsy-related complications are common, such as pneumothorax and hemoptysis (7).

Liquid biopsy is emerging as an important clinical tool and has significant potential to offer a supplement or a conditional alternative to tissue biopsy for tumor genotyping (6). Liquid biopsy offers the advantages of being non-invasive, easily accessible, and can be performed repeatedly (8). Presently, cell-free DNA is available for liquid biopsy (9). Mature testing platforms of EGFR mutation include next generation sequencing technologies (NGS), digital platforms [droplet digital PCR (ddPCR), Beads, Emulsions, Amplification, and Magnetic (BEAMing)] and real time PCR [Cobas, Amplification Refractory Mutation System (ARMS-PCR)]. Thress et al. demonstrated that the Cobas EGFR Mutation Test and BEAMing dPCR had high sensitivity (82–87%) and specificity (97%) for EGFR-sensitizing mutations (10). Moreover, Feng et al. indicated that the sensitivity of ddPCR was similar with ARMS in plasma EGFR detection (80.4 vs. 76.5%), as was the specificity (89.3 vs. 100%) (11). These findings showed the high sensitivity and specificity of PCR platforms, suggesting that EGFR mutations can be accurately detected in cfDNA. In addition, the PCR-based methods had the advantages of being both rapid and inexpensive, and suitable for detection of specific point mutations (12).

Several studies have reported promising results detecting EGFR mutation from cfDNA of patients with lung cancer using ddPCR and ARMS-PCR (11, 13–15). The question of interest is whether these tissue-free methods are sufficiently accurate to be considered a supplement or even alternative to tissue genotyping. Therefore, we conducted this meta-analysis to determine the diagnostic accuracy of the ddPCR system and the ARMS-PCR system for detecting EGFR mutation in cfDNA, using tissue biopsy results as the standard detection modality.

## Materials and Methods

This meta-analysis was conducted according to the PRISMA Checklist.

### Literature and Search Strategy

All potentially relevant studies were retrieved through search of PubMed, Web of science, Embase and the Cochrane library databases up to Dec 1, 2019, using a combination of key words “lung cancer,” “EGFR,” “droplet digital PCR,” and “amplification refractory mutation system PCR.” No search limitations were set. The previous published articles and reviews were inspected to identify studies not included by the initial search. This study is registered with PROSPERO, number CRD42019120049.

### Inclusion and Exclusion Criteria

Eligible studies should meet the following criteria: (i) included patients with lung cancer diagnosed by histopathology or cytologically; (ii) studied diagnostic accuracy of ddPCR or ARMS-PCR for detecting EGFR sensitivity mutation based on cfDNA or ctDNA; (iii) EGFR mutation statuses were compared with tumor tissues.

Studies were excluded if (i) data was insufficient to calculate the sensitivity or specificity for this meta-analysis, (ii) they were review articles, abstracts, case reports, commentary articles, editorials, expert opinions, non-comparative studies, letters, unrelated to research topics, or duplicate reports.

### Data Extraction

Data were extracted independently by two reviewers (Li C.C. and Liang H.R.), and conflicts were resolved by a third reviewer (He Q.H.). For the selected studies, the name of first author, year of publication, country of origin, sample size, basic characteristics of studied population, clinical stage, tumor histology, percentage of TKI-naïve, and TKI-treated patients, techniques used for EGFR mutation detection for both tissue sample and cfDNA, true positive (TP), false positive (FP), false negative (NP), and true negative (TN) were collected from eligible studies. Subgroup analyses, and comparison of two PCR platforms were conducted according to EGFR mutation type, tumor stage and EGFR-TKI treatment, respectively.

### Quality Assessment

Quality assessment of diagnostic accuracy studies 2 (QUADAS-2) is a tool used to evaluate the quality of primary diagnostic accuracy studies, including patient selection, index tests, reference standard, and flow and timing. QUADAS-2 was evaluated by two reviewers (CL and HL).

### Statistical Analysis

Sensitivity, specificity, diagnostic odds ratio (DOR) and the area under the summary receiver-operating characteristic curve (AUROC) were pooled. The value of DOR is calculated by the positive likelihood ratio (PLR)/the negative likelihood ratio (NLR), and its value ranges from 0 to infinity, with higher value indicating better discriminatory test performance.

We use Cochrane's Q and the *I*^2^ statistic to examine the heterogeneity. *P* ≤ 0.05 and *I*^2^ ≥ 50% mean that significant heterogeneity existed in pooled statistics. In addition, publication bias was detected by the Deek's funnel plot asymmetry test and *P* < 0.05 indicated the presence of publication bias.

The analysis was performed with STATA 13.0 software (STATA corporation, College Station, TX, USA) with the MIDAS module and Meta-Disc 1.4 (Ramón y Cajal Hospital in Madrid, Spain).

## Results

### Study Selection and Quality Assessment

**Figure 1:**
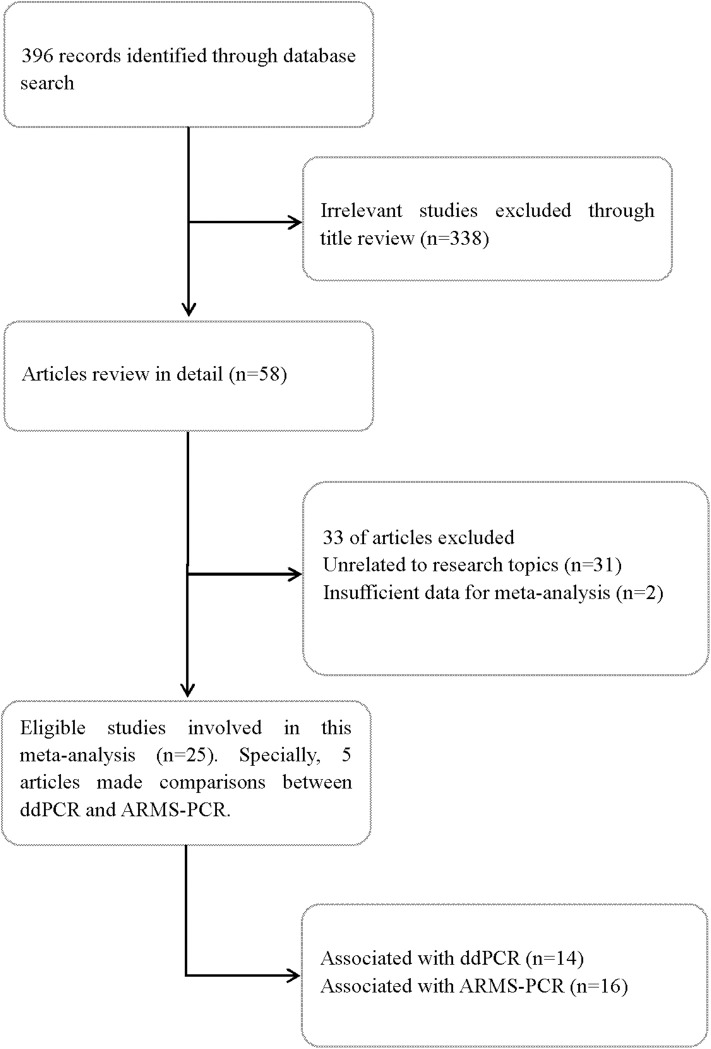
Flow diagram detailing the search strategy of the included studies in this meta-analysis.

A total of 396 records were screened according to the search strategy up to Dec 1, 2019. Finally, 25 full-text articles were identified and reviewed. Of the included articles, 14 studied ddPCR (10, 11, 15–26) and 16 studied ARMS-PCR (10, 11, 13–15, 20, 22, 27–35). Specifically, five articles made direct comparisons between ddPCR and ARMS-PCR (10, 11, 15, 20, 22). Figure 1 summarized the flow chart. The quality assessment of each study is summarized in Table S1.

### Characteristics of Included Studies

Twenty-five studies involving 4,881 cases were identified and included for analysis. The majority of patients were Asians with advanced NSCLC. To assess ddPCR performance of cfDNA-based EGFR mutation detection, a total of 1,105 samples were used for testing EGFR mutation and compared with the result of tissue biopsy. Similarly, a total of 3,950 samples were tested and compared with tissue biopsy to assess diagnostic performance of ARMS-PCR. Table 1 summarized the characteristics of all relevant studies. It should be noted that, some studies only presented the results of mutation in exon 19 deletion and L858R, rather than total mutations level of EGFR. Therefore, we added the sample number of mutations of exon 19 deletion and L858R together to get an overall result of EGFR mutation in each study.

**Table 1:** Characteristics of included studies.

**Study**	**Country**	**Sample size**	**Age**	**Female (%)**	**Smoker (%)**	**Histology**	**Clinical stage**	**TKI naïve (%)**
Ishii et al. (16)	Japan	18	50-81	89	6	NSCLC	Recurrence	0
Lee et al. (18)	Korea	81	32-81	62	37	NSCLC	IV/recurrence	0
Sacher et al. (19)	US	180	18+	62	NA	NSCLC	IIIB/IV/recurrence	0, 100^†^
Thress et al. (10)	UK	38	NA	NA	NA	NSCLC	Advanced	0
Feng et al. (11)	China	79	30–75	54	32	NSCLC	Advanced	100
Xu et al. (15)	China	20	37–76	50	30	NSCLC	I–IV	40
Zhang et al. (22)	China	122	30–85	47	42	NSCLC	III–IV	100
Wang et al. (20)	China	65	32–85	38	48	LC	I—IV/uncertain	NA
Yu et al. (21)	China	22	35–74	54	NA	NSCLC	IIIB–IV	86
Zhang et al. (23)	China	215	NA	41	44	NSCLC	IIIB–IV	100
Zhu et al. (17)	China	86	28–81	35	NA	NSCLC	IIIB–IV	100
Zhu et al. (24)	China	51	60.89 ± 1.48	39	55.8	NSCLC	I–IV	56.9
Li et al. (32)	China	109	NA	53	33	NSCLC	IIIB–IV	96.3
Cui et al. (34)	China	180	37–77	48	NA	NSCLC	IIIB–IV	70
Douillard et al. (14)	France	1060	32–82	71	39	LC	IIIA–IV	100
Duan et al. (30)	China	94	58 ± 11	35	51	LC	IIA–IV	100
Li et al. (29)	China	164	32–81	41.5	48.8	LC	IIB–IV/recurrence	58.5
Liu et al. (28)	China	86	28–81	35	55	NSCLC	IIIB–IV	NA
Ma et al. (31)	China	219	26–81	34	49	LC	IIIA–IV	100
Su et al. (35)	China	107	29–81	58	15	NSCLC	I–IV	73.8
Wan et al. (13)	China	2463	NA	NA	NA	LC	I–IV	0
Xu et al. (27)	China	51	25–77	39	37	NSCLC	IIIB–IV	100
Zhou et al. (33)	China	447	27–86	45	47	LC	I–IV	98.2
Jiang et al. (25)	China	50	NA	NA	NA	NSCLC	NA	100
Guo et al. (26)	China	201	NA	52.2	NA	NSCLC	I–IV	66.2

† *Patients of this study have divided into two groups according to TKI used. TKI, tyrosine kinase inhibitors; LC, lung cancer; NSCLC, non-small-cell lung cancer*.

### Overall Accuracy of the ddPCR and ARMS-PCR Test

**Figure 2:**
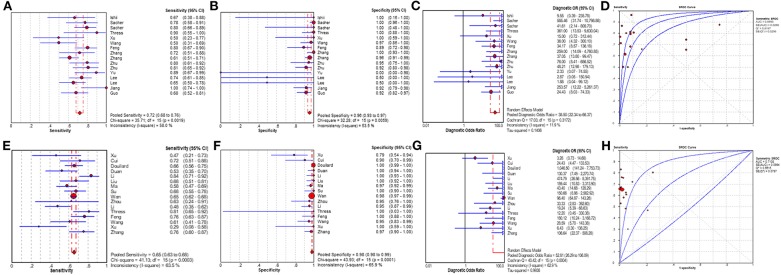
The results of meta-analysis. **(A)** sensitivity, **(B)** specificity, **(C)** diagnostic odds ratio, and **(D)** SROC curve for ddPCR; **(E)** sensitivity, **(F)** specificity, **(G)** diagnostic odds ratio, and **(H)** SROC curve for ARMS-PCR. Two articles of ddPCR had two status including prior treatment group and disease progression group. Add 0.5 to all cells of the studies with zero.

**Figure 3:**
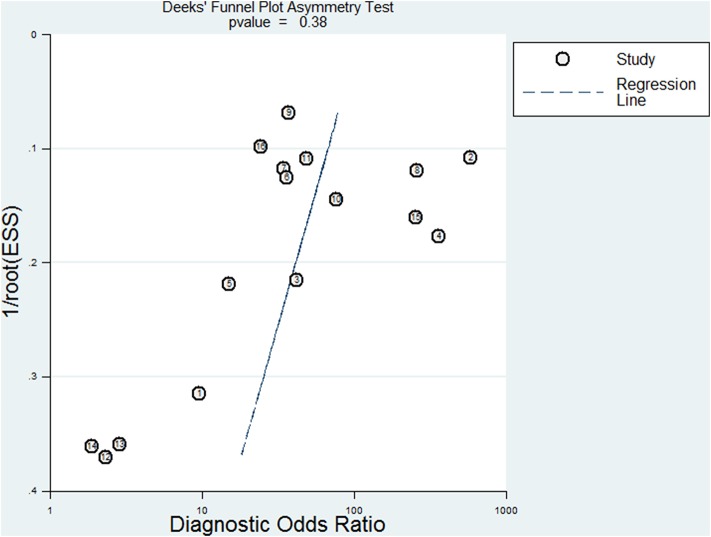
Assessment of publication bias by Deek's funnel plot asymmetry test in ddPCR system.

The plasma testing sensitivity, specificity, DOR, and AUROC, compared with the matched tumor tissues, were 72.1% (95% CI, 68.2–75.7%), 95.6% (95% CI, 93.5–97.1%), 38.5 (95% CI, 22.3–66.4), 0.89 (95% CI, 0.83–0.95) for ddPCR, and 65.3% (95% CI, 62.9–67.6%), 98.2% (95% CI, 97.6–98.7%), 52.8 (95% CI, 26.3–106.1), 0.71 (95% CI, 0.52–0.91) for ARMS-PCR, respectively (Figure 2). There was no publication bias for outcome measures with asymmetrical appearance on funnel plot analysis (Figure 3), and *P* > 0.05 in Deek's test.

### Comparison of ddPCR and ARMS-PCR in Different Subgroups

Subsequently, results of the two platforms in different EGFR-sensitizing mutations, tumor stages and EGFR-TKI treatment status were assessed by stratified analysis (Table 2). Significant difference in sensitivity was found between tumor stage subgroups (IIIB–IV subgroup vs. IA–IV subgroup) in ARMS-PCR (73.7 vs. 64.2%, *P* = 0.008), but not in ddPCR (72.5 vs. 71.2%, *P* = 0.756).

**Table 2:** The results of meta-analysis.

**Index method**	**Included studies**	**Sensitivity**	**I^**2**^**	***P***	**Specificity**	***I*^**2**^**	***P***	**DOR**	***I*^**2**^**	***P***	**AUROR**
ddPCR^†^	14	72.1% (95% CI, 68.2–75.7%)	58%	0.002	95.6% (95% CI, 93.5–97.1%)	53.5%	0.006	38.5 (95% CI, 22.3–66.4)	11.9%	0.317	0.89 (95% CI, 0.83–0.95)
ARMS-PCR^†^	16	65.3% (95% CI, 62.9–67.6%)	63.5%	0.001	98.2% (95% CI, 97.6–98.7%)	65.9%	0.001	52.8 (95% CI, 26.3–106.1)	62.9%	0.001	0.71 (95% CI, 0.52–0.91)
**SUBGROUP ANALYSIS OF EXON 19 DELETION IN TWO SYSTEMS**^**‡**^											
ddPCR	11	72.9% (95% CI, 67.2–78.2%)	46.0%	0.035	99.1% (95% CI, 98.2–99.7%)	18.0%	0.262	179.6 (95% CI, 85.9–375.5)	0%	0.997	0.97 (95% CI, 0.94–1.00)
ARMS-PCR	11	66.3% (95% CI, 60.9–71.3%)	44.3%	0.056	99.3% (95% CI, 98.6–99.7%)	68.9%	0.001	113.7 (95% CI, 39.9–323.4)	53.4%	0.018	0.65 (95% CI, 0.25–1.00)
**SUBGROUP ANALYSIS OF L858R IN TWO SYSTEMS**^**§**^											
ddPCR	12	69.7% (95% CI, 63.3–75.5%)	45.6%	0.032	98.2% (95% CI, 97.0–99.0%)	31.3%	0.125	96.5 (95% CI, 53.2–175.2)	0%	0.872	0.96 (95% CI, 0.91–1.00)
ARMS-PCR	11	61.6% (95% CI, 54.9–68.0%)	51.1%	0.025	99.3% (95% CI, 98.7–99.7%)	33.8%	0.128	110.1 (95% CI, 49.7–243.8)	26.3%	0.193	0.79 (95% CI, 0.42–1.00)
**SUBGROUP ANALYSIS OF STAGE IIIB**–**IV AND RECURRENCE IN TWO SYSTEMS**											
ddPCR^∧^	8	73.4% (95% CI, 68.8–77.6%)	47.3%	0.048	95.7% (95% CI, 92.9–97.6%)	62.7%	0.004	34.4 (95% CI, 15.3–77.3)	27.5%	0.191	0.85 (95% CI, 0.75–0.95)
ARMS-PCR	6	62.9% (95% CI, 55.9–69.6%)	80.9%	0.001	95.6% (95% CI, 91.9–98.0%)	70.4%	0.005	24.5 (95% CI, 6.1–98.1)	65.0%	0.014	0.69 (95% CI, 0.16, 1.00)
**SUBGROUP ANALYSIS OF STAGE IA**–**IV IN TWO SYSTEMS**											
ddPCR^∧^	3	64.7% (95% CI, 55.2–73.3%)	59.5%	0.060	94.4% (95% CI, 88.9–97.7%)	0%	0.538	29.8 (95% CI, 12.6–70.6)	0%	0.79	0.92 (95% CI, 0.77–1.00)
ARMS-PCR^∧^	4	65.1% (95% CI, 62.2–68.0%)	0%	0.929	98.0% (95% CI, 97.2–98.7%)	13.9%	0.323	88.7 (95% CI, 60.9–129.2)	0%	0.419	0.49 (95% CI, 0–1.00)
**SUBGROUP ANALYSIS OF TKI-NAIVE IN TWO SYSTEMS**^**¶**^											
ddPCR^∧^	6	72.7% (95% CI, 66.6–78.2%)	69.1%	0.006	96.6% (95% CI, 94.1–98.2%)	65.9%	0.012	62.7 (95% CI, 28.1–140.1)	14.3%	0.323	0.94 (95% CI, 0.87–1.00)
ARMS-PCR^∧^	6	64.5% (95% CI, 59.1–69.6%)	56.8%	0.041	98.7% (95% CI, 97.7–99.4%)	83.1%	0.001	74.5 (95% CI, 14.9–373.8)	79.5%	0.001	0.59 (95% CI, 0.22–0.97)
**SUBGROUP ANALYSIS OF TKI-TREATED IN TWO SYSTEMS**											
ddPCR	8	75.6% (95% CI, 69.0–81.5)	45.2%	0.090	93.5% (95% CI, 88.4–96.8%)	48.1%	0.073	29.7 (95% CI, 14.3–61.4)	0%	0.462	0.87 (95% CI, 0.79–0.96)
ARMS-PCR	8	65.5% (95% CI, 62.8–68.1%)	75.9%	0.001	98.0% (95% CI, 97.2–98.6%)	24.3%	0.235	45.4 (95% CI, 18.9–108.9)	48.3%	0.060	0.86 (95% CI, 0.66–1.00)

† Specially, five articles made direct comparisons between ddPCR and ARMS-PCR;

‡ Two articles of ddPCR did not give the results of exon 19 deletion mutation or L858R, separately; five articles of ARMS-PCR did not give the results of exon 19 deletion mutation or L858R, separately;

§ One article of ddPCR only presented L858R data;

∧ Three article of ddPCR and six articles of ARMS-PCR did not meet the requirements of subgroup analysis, separately.

¶ *One article of ddPCR and two articles of ARMS-PCR did not give the results of TKI used, separately. DOR, diagnostic odds ratio; AUROC, area under the summary receiver-operating characteristic curve; ddPCR, droplet digital PCR; ARMS-PCR, amplification refractory mutation system PCR; L858, exon 21 Leu858Arg; TKI, tyrosine kinase inhibitors*.

### Indirect and Direct Comparison of ddPCR and ARMS-PCR

Twenty five full-text articles were included in the indirect comparison and the detailed characteristics of clinical stage of the enrolled patients are summarized in Table S2. In studies indirectly comparing the two PCR systems, there was a significant difference in sensitivity (*P* = 0.003) and in specificity (*P* = 0.007) (Table 3). We performed indirect comparison about sensitivity between ddPCR and ARMS-PCR systems, better sensitivities for ddPCR were observed in stage IIIB–IV (73.4 vs. 62.9%, *P* = 0.012), TKI-naïve (72.7 vs. 64.5%, *P* = 0.040), and TKI-treated (75.6 vs. 65.5%, *P* = 0.035) patients. Compared to ARMS-PCR, more favorable specificity was found in the TKI-treated subgroup using ddPCR (93.5 vs. 98.0%, *P* = 0.038). In studies simultaneously comparing the two platforms, however, we observed no significant difference in specificity (97.3 vs. 98.7%, *P* = 0.473) and sensitivity (69.3 vs. 69.0%, *P* = 0.960) between ddPCR and ARMS-PCR, regardless of EGFR mutation type and EGFR-TKI treatment.

**Table 3:** The results of direct and indirect comparison of ddPCR and ARMS-PCR.

**Results of comparison**		**Platforms**	***P*-value**
Overall results	Direct comparison	Sensitivity: ddPCR vs. ARMS-PCR = 69.3 vs. 69.0%	0.960
		Specificity: ddPCR vs. ARMS-PCR = 97.3 vs. 98.7%	0.473
	Indirect comparison	Sensitivity: ddPCR vs. ARMS-PCR = 72.1 vs. 65.3%	0.003
		Specificity: ddPCR vs. ARMS-PCR = 95.6 vs. 98.2%	0.007
Subgroup results	Direct comparison under the same platforms	Sensitivity: ddPCR (19del) vs. ddPCR (L858R) = 73.2 vs. 68.4%	0.282
		Specificity: ddPCR (19del) vs. ddPCR (L858R) = 99.3 vs. 98.8%	0.430
		Sensitivity: ddPCR (IIIB–IV) vs. ddPCR (IA–IV) = 72.5 vs. 71.2%	0.756
		Specificity: ddPCR (IIIB–IV) vs. ddPCR (IA–IV) = 93.5 vs. 98.8%	0.010
		Sensitivity: ddPCR (TKI-naive) vs. ddPCR (TKI-treated) = 71.7 vs. 74.1%	0.567
		Specificity: ddPCR (TKI-naive) vs. ddPCR (TKI-treated) = 96.5 vs. 95.4%	0.733
		Sensitivity: ARMS-PCR (19del) vs. ARMS-PCR (L858R) = 66.3 vs. 61.6%	0.271
		Specificity: ARMS-PCR (19del) vs. ARMS-PCR (L858R) = 99.3 vs. 99.3%	1.000
		Sensitivity: ARMS-PCR (IIIB–IV) vs. ARMS-PCR (IA–IV) = 73.7 vs. 64.2%	0.008
		Specificity: ARMS-PCR (IIIB–IV) vs. ARMS-PCR (IA–IV) = 96.3 vs. 98.3%	0.234
		Sensitivity: ARMS-PCR (TKI-naive) vs. ARMS-PCR (TKI-treated) = 63.9 vs. 65.4%	0.766
		Specificity: ARMS-PCR (TKI-naive) vs. ARMS-PCR (TKI-treated) = 96.7 vs. 98.5%	0.112
	Indirect comparison under the different platforms	Sensitivity: ddPCR (19del) vs. ARMS-PCR (19del) = 72.9 vs. 66.3%	0.087
		Specificity: ddPCR (19del) vs. ARMS-PCR (19del) = 99.1 vs. 99.3%	0.673
		Sensitivity: ddPCR (L858R) vs. ARMS-PCR (L858R) = 69.7 vs. 61.6%	0.080
		Specificity: ddPCR (L858R) vs. ARMS-PCR (L858R) = 98.2 vs. 99.3%	0.053
		Sensitivity: ddPCR (IIIB–IV) vs. ARMS-PCR (IIIB–IV) = 73.4 vs. 62.9%	0.012
		Specificity: ddPCR (IIIB–IV) vs. ARMS-PCR (IIIB–IV) = 95.7 vs. 95.6%	0.959
		Sensitivity: ddPCR (IA–IV) vs. ARMS-PCR (IA–IV) = 64.7 vs. 65.1%	0.934
		Specificity: ddPCR (IA–IV) vs. ARMS-PCR (IA–IV) = 94.4 vs. 98.0%	0.114
		Sensitivity: ddPCR (TKI-naive) vs. ARMS-PCR (TKI-naive) = 72.7 vs. 64.5%	0.040
		Specificity: ddPCR (TKI-naive) vs. ARMS-PCR (TKI-naive) = 96.6 vs. 98.7%	0.064
		Sensitivity: ddPCR (TKI-treated) vs. ARMS-PCR (TKI-treated) = 75.6 vs. 65.5%	0.035
		Specificity: ddPCR (TKI-treated) vs. ARMS-PCR (TKI-treated) = 93.5 vs. 98.0%	0.038

*ddPCR, droplet digital PCR; ARMS-PCR, amplification refractory mutation system PCR; 19del, exon 19 deletion; L858, exon 21 Leu858Arg; TKI, tyrosine kinase inhibitors*.

## Discussion

Precise detection of EGFR mutation in lung cancer can allow clinicians to assign patients to highly specific treatment, especially for those with EGFR-sensitizing mutations as a series of clinical trials has proven (4, 5). Many retrospective studies have reported that patients with ctDNA-based EGFR mutation status have better clinical outcomes with EGFR-TKIs than those without EGFR mutation (14, 36). In a prospective clinical trial reported by Wang et al., detection of EGFR mutation in ctDNA was a selection method to provide patients with appropriate first-line gefitinib treatment, providing more evidence to guide treatment decisions for those patients with advanced lung cancer who cannot obtain tumor tissue samples (37). It is interesting and meaningful to answer whether these tissue-free methods are sufficiently accurate to be considered a supplement or even alternative to tissue genotyping. Accordingly, this meta-analysis was conducted to assess the diagnostic accuracy of ddPCR system and ARMS-PCR system for detecting EGFR mutation in cfDNA.

In this meta-analysis, using tissue test as reference, we found that both ddPCR and ARMS-PCR had high diagnostic accuracy when testing in plasma cfDNA. By direct comparison, there was no significant difference between ddPCR and ARMS-PCR in overall accuracy. However, significant difference could be found in sensitivity and in specificity by indirect comparison. The direct comparison of results of the two platforms reported here suggested ddPCR had a higher sensitivity than ARMS-PCR in subgroup analysis of stage IA–IV. Combining the result of stratified analysis of tumor stage in sensitivity in ARMS-PCR, which demonstrated that ARMS-PCR had a higher sensitivity in the pure advanced lung cancer subgroup compared with early stage patients. We suspected that the sensitivity of ddPCR might be higher than ARMS-PCR in early stages, which warrants more data specific to early stage lung cancers. After indirect comparison, significant difference was also found in sensitivity between ddPCR and ARMS-PCR in the IIIB–IV subgroup, TKI-naïve subgroup and TKI-treated subgroup. Obvious higher specificity for ARMS-PCR was also observed in the TKI-treated subgroup. The indirect comparison of results of the two platforms suggested ddPCR had a higher sensitivity and ARMS-PCR had a higher specificity in some situations. However, the above results showed the discordance of sensitivity and specificity in two PCR platforms. The difference of results between direct and indirect comparison may be caused by insufficient sample sizes as only five articles had data for direct comparison. Furthermore, studies demonstrated the sensitivity of ARMS-PCR was 0.1% (38) and the sensitivity of ddPCR was 0.001% (39) detected in plasma, ddPCR showed improved limits of detection compared to ARMS-PCR, which may give rise to diverse results. Studies also indicated the abundance of ctDNA in patients with advanced stage varied from 0.1 to 53.2%, and was lower (< 0.01%) in patients with early stage cancer (40, 41). Thus, it may sometimes show different diagnostic results in ddPCR and AMRS-PCR. In addition, by stratified analysis of EGFR-sensitizing mutations, we found that the exon 19 deletion testing sensitivity seemed higher than L858R in both testing systems, this is probably because tumor mutation burden (TMB) or ctDNA in plasma from the tumor of exon 19 deletion was higher than L858R, resulting in an increase of cfDNA in plasma.

Based on the excellent diagnostic performances of ddPCR and ARMS-PCR, we have reason to believe that it is rational to use these tissue-free methods as a supplement or an alternative option to tissue genotyping. Of note, both methods are relatively quick and inexpensive to detect the allelic frequency of mutations in cfDNA, but they cannot provide a comprehensive molecular profile of cancer. Besides, the sensitivity of the PCR systems could be limited if the proportion of tumor DNA in cfDNA is low. Owing to the high specificity, a patient with a negative result due to low percentage of mutant cfDNA could retest or the diagnosis could be determined in other ways. When a positive result is found, the patient may receive EGFR-TKI therapy, and should be followed up to evaluate the therapeutic effect. In addition, to validate the effectiveness and accuracy of liquid biopsy, prospective study based on the above test platforms for detecting EGFR mutation in cfDNA to compare lung cancer patients with healthy people as control is required and necessary, so as to set up an optimal cut-off point and reduce false positives. At the same time, we can also increase sample sizes to identify the diagnostic accuracy of ddPCR and ARMS-PCR.

To date, liquid biopsy is a complement to the tissue biopsy. If we want to use the result of genotyping of liquid biopsy directly in patients whose tissue samples are not available, we need to focus on the result of specificity first. When the specificity of liquid biopsy is increased to be consistent with tissue biopsy, it is reasonable to use liquid biopsy as an alternative to tissue biopsy in clinical practice for genotyping. For this reason, we should be cautious of false positives, though research has reported that cfDNA analysis does not involve formaldehyde fixation which can reduce false positive results due to deamination (42). The reasons for false positives can be divided into detection causes and non-detection causes. Detection causes mainly include: (i) Determination of cut-off values for EGFR mutations that were defined too low, (ii) Single tissue biopsy specimens were difficult to reflect the genetic characteristics of the whole tumor on intratumor heterogeneity, which meant even the result of cfDNA may be correct sometimes, false positive results by tissue biopsy conduced an opposite conclusion, (iii) Non-specific annealing of PCR primers could result in a false positive when the concentration of wild-type template was much higher than mutant template (20). Furthermore, the time interval between tissue samples acquired first and plasma samples acquired later may also cause false positives due to the tumor burdens becoming more severe as the disease progressed. Non-detection causes are mainly reflected in the following: (i) germline mutation, (ii) non-tumorous EGFR mutation, (iii) subclone EGFR. Germline mutation was caused by the change of family gene, contributing to the generation of family background in this population, which led to false positive results. The incorrect results of non-tumorous EGFR mutation were similar to germline mutation. If liquid biopsy technology can reduce false positive and increase specificity further, it would greatly benefit, not only tissue genotyping but also the longitudinal surveillance of clonal evolution (43).

We acknowledge several limitations to our study. First, it should be noted that, some studies only presented the results of mutations in exon 19 deletion and L858R, rather than total mutation level of EGFR. Therefore, we added the sample number of mutations of exon 19 deletion and L858R together to get an approximate result of EGFR mutation in each study. Second, most publications were retrospective studies, which may improve diagnostic accuracy artificially by setting a cut-off value, prospective clinical trials are needed for further investigation. Third, the diagnostic methods of ddPCR and ARMS-PCR, such as different extraction methods of DNA, and different types of primers and probes were not analyzed in this study. Last but not the least, in studies directly comparing the two PCR systems, the sample size was not large enough and the literature reports were limited. Through indirect comparison we were able to overcome sample selection bias to some extent however, the power of the test was not strong enough.

## Conclusion

This study demonstrates that both ddPCR and ARMS-PCR have a high specificity with a practical sensitivity for detecting EGFR mutation in cfDNA of advanced lung cancer patients, which supports their application as a supplement or a conditional-alternative to tissue biopsy in clinical practice for genotyping. In addition, ddPCR-based plasma genotyping may be applied in clinical use more often with minimal false positives.

## Data Availability Statement

The raw data supporting the conclusions of this article will be made available by the authors, without undue reservation, to any qualified researcher.

## Author Contributions

WL, JH, and CL conceived the study, and take responsibility for the integrity of the data and accuracy of the data analysis. QH and HL did the literature research, performed study selection, data extraction, and synthesis. BC, JL, and YZ participated in the analysis and interpretation of the data. SX, MG, and ZL wrote the draft review paper. WL, ZL, and CL revised the manuscript critically for important intellectual content and redrafted some of its section. All authors contributed to manuscript revision, read and approved the submitted version.

### Conflict of Interest

The authors declare that the research was conducted in the absence of any commercial or financial relationships that could be construed as a potential conflict of interest.
