# Supplementary material for: Diagnostic Accuracy of Droplet Digital PCR and Amplification Refractory Mutation System PCR for Detecting EGFR Mutation in Cell-Free DNA of Lung Cancer: A Meta-Analysis
Source: Front Oncol. 2020 Mar 3;10:290. doi: 10.3389/fonc.2020.00290 (PMC7063461; doi:10.3389/fonc.2020.00290)
Supplement: Supplementary file 1 [file Table_1.pdf]

Table S1 Quality assessment of included studies

Study	Risk of bias				Applicability concerns		
	Patient selection	Index text	Reference methods	Flow and timing	Patient selection	Index text	Reference methods
Ishii	**L**	**L**	**L**	**L**	**L**	**L**	**L**
Lee	**L**	**?**	**L**	**L**	**L**	**L**	**L**
Sacher	**L**	**?**	**L**	**L**	**L**	**L**	**L**
Thress	**L**	**?**	**L**	**H**	**L**	**L**	**L**
Feng	**L**	**L**	**L**	**L**	**L**	**L**	**L**
Xu	**L**	**?**	**L**	**L**	**L**	**L**	**L**
Zhang	**L**	**L**	**L**	**L**	**L**	**L**	**L**
Wang	**L**	**L**	**L**	**L**	**L**	**L**	**L**
Yu	**L**	**L**	**L**	**L**	**L**	**L**	**L**
Zhang	**L**	**L**	**L**	**L**	**L**	**L**	**L**
Zhu	**L**	**L**	**L**	**L**	**L**	**L**	**L**
Zhu	**L**	**L**	**?**	**L**	**L**	**L**	**L**
Li	**L**	**L**	**L**	**L**	**L**	**L**	**L**
Cui	**L**	**?**	**L**	**L**	**L**	**L**	**L**
Douillard	**L**	**?**	**L**	**L**	**L**	**L**	**L**
Duan	**L**	**?**	**L**	**L**	**L**	**L**	**L**
Li	**L**	**L**	**L**	**L**	**L**	**L**	**L**
Liu	**L**	**?**	**L**	**L**	**L**	**L**	**L**

Table S1 Continued

Study	Risk of bias				Applicability concerns		
	Patient selection	Index text	Reference methods	Flow and timing	Patient selection	Index text	Reference methods
Ma	**L**	**?**	**L**	**L**	**L**	**L**	**L**
Su	**L**	**?**	**L**	**L**	**L**	**L**	**L**
Wan	**L**	**?**	**L**	**L**	**L**	**L**	**L**
Xu	**L**	**?**	**L**	**L**	**L**	**L**	**L**
Zhou	**L**	**?**	**L**	**L**	**L**	**L**	**L**
