# Supplementary material for: Diagnostic Accuracy of Droplet Digital PCR and Amplification Refractory Mutation System PCR for Detecting EGFR Mutation in Cell-Free DNA of Lung Cancer: A Meta-Analysis
Source: Front Oncol. 2020 Mar 3;10:290. doi: 10.3389/fonc.2020.00290 (PMC7063461; doi:10.3389/fonc.2020.00290)
Supplement: Supplementary file 2 [file Table_2.pdf]

Supplementary Table 2. Other detailed clinical characteristics of the enrolled patients.

Study	Year	I	II	III	IV	Recurrence	Total
Ishii	2015	0	0	0	0	18	18
Lee	2016	0	0	0	68	13	81
Sacher	2016	0	0	3	172	5	180
Thress	2015	N/A	N/A	N/A	N/A	N/A	N/A
Feng	2018	N/A	N/A	N/A	N/A	N/A	N/A
Xu	2017	5	2	6	7	0	20
Zhang	2017	0	0	43	79	0	122
Wang	2017	2	0	14	46	0	62
Yu	2017	0	0	4	18	0	22
Zhang	2017	0	0	36	179	0	215
Zhu	2015	0	0	4	82	0	86
Zhu	2017	2	0	4	44	0	50
Li	2017	0	0	20	89	0	109
Cui	2017	0	0	21	159	0	180
Douillard	2014	N/A	N/A	N/A	N/A	N/A	N/A
Duan	2015	0	1	13	80	0	94
Li	2014	0	0	14	131	19	164
Liu	2013	0	0	4	82	0	86
Ma	2016	0	0	48	171	0	219
Su	2018	2	7	18	80	0	107
Wan	2017	N/A	N/A	N/A	N/A	N/A	N/A
Xu	2012	0	0	6	45	0	51
Zhou	2017	50	22	72	303	0	447
Jiang	2019	N/A	N/A	N/A	N/A	N/A	N/A
Guo	2019	N/A	N/A	N/A	172	0	201
